# Ethnic inequality, multimorbidity and psychosis: can a syndemic framework resolve disputed evidence?

**DOI:** 10.1038/s41537-023-00367-8

**Published:** 2023-06-09

**Authors:** Uzma Zahid, Georgina M. Hosang, Daniela Fonseca de Freitas, Roisin Mooney, Kamaldeep Bhui

**Affiliations:** 1 Department of Psychiatry, University of Oxford, Oxford, UK; 2 Department of Psychology, Institute of Psychiatry, Psychology & Neuroscience, King’s College London, London, UK; 3 Centre for Psychiatry and Mental Health, Wolfson Institute of Population Health, Queen Mary University of London, London, UK; 4 Department of Psychological Medicine, Institute of Psychiatry, Psychology & Neuroscience, King’s College London, London, UK; 5 Nuffield Department of Primary Care Health Sciences, Wadham College, University of Oxford, Oxford, UK; 6 Queen Mary University London Global Policy Institute, London, UK

**Keywords:** Psychosis, Schizophrenia

## Abstract

Syndemic theory is described as population-level clustering or co-occurrence of health conditions in the context of shared aetiologies that interact and can act synergistically. These influences appear to act within specific places of high disadvantage. We suggest ethnic inequality in experiences and outcomes of multimorbidity, including psychosis, may be explained through a syndemic framework. We discuss the evidence for each component of syndemic theory in relation to psychosis, using psychosis and diabetes as an exemplar. Following this, we discuss the practical and theoretical adaptations to syndemic theory in order to apply it to psychosis, ethnic inequality and multimorbidity, with implications for research, policy, and practice.

## Introduction

Psychotic illnesses such as schizophrenia are characterised by hallucinations, delusions, negative symptoms (e.g., social withdrawal, lack of motivation), and cognitive disorganisation^1^. Approximately 6% of the population experience psychosis-like experiences^2^ (that do not meet the threshold for a diagnosis of a psychotic disorder), and 1% are diagnosed with psychotic disorders, such as schizophrenia^3^. A higher incidence of psychoses is observed among ethnic minority communities, migrants^4^, urban dwellers^5^ and those who experience multiple disadvantages in terms of poverty and deprivation^6^. Individuals with psychotic illnesses are likely to have poor social relationships^7^ and diminished quality of life^8^ compared to the general population. Furthermore, psychosis is associated with considerable morbidity and mortality. Up to 75% of people with a psychotic disorder also meet the criteria for a physical illness^9^ and 57.3% with another psychiatric condition^10^. Psychiatric and medical comorbidities are the rule rather than the exception. Such comorbidities are problematic in terms of treatment complications but can be lethal since they are associated with premature mortality of up to 15 years^11^.

A broad range of factors are associated with the development of psychosis as well as multimorbidity, spanning bio-psycho-social models^12^. However, prevention and intervention efforts along these lines have not been effective for all people with psychosis especially marginalised groups, including ethnic minorities. Place-based and ethnic inequalities in the incidence of psychosis appear to be driven by social and environmental influences rather than genetics or biology^13^. The aim of this paper is to assess the applicability of syndemic theory when applied to ethnic inequalities in people living with psychosis and facing multiple disadvantages and poor health outcomes.

## Syndemic theory

The term syndemic was first described by Singer^14^ as population-level clustering of social and health problems. He described “SAVA”, the interacting epidemics of substance abuse, violence, and HIV/AIDS in an inner-city Hispanic population in the USA that were intertwined and mutually reinforcing one another in the local context. Singer argued that the local epidemics of HIV and substance use had to be considered in concert because the pathways of transmission were inextricably linked; they existed in a particular socio-economic context and were deepened by structural violence. Therefore, it was important to recognise the linkages between various health and social conditions when considering interventions. Syndemic theory has been useful in the past in several ways, including understanding complex health issues. By recognising the syndemic nature of a health issue, researchers and practitioners can better understand the underlying social, economic, and environmental factors that contribute to the problem and develop more effective interventions. Syndemic theory emphasises the need for comprehensive interventions that address all aspects of a health issue rather than just treating individual diseases or conditions. By addressing the syndemic, interventions can be developed that are more effective in improving health outcomes. Indeed, the SAVA syndemic framework is now widely used to develop interventions in the field of HIV.

In psychosis, existing theories often overlook the interactions between individual contexts (e.g., social, political and ecological factors) and histories. In contrast, syndemic theory assumes all these domains are interconnected and clustered in certain populations and places. The syndemic framework emphasises the importance of both macro (e.g., discrimination) and micro (e.g., interpersonal stressors) level factors when examining the origins of co-occurring health conditions, which can be accounted for when designing public health interventions or implementing changes to health services.

In the literature, there are slight variations in syndemic criteria, but the essential elements producing negative health outcomes are as follows:

1. Two or more diseases or health conditions clustering within a particular population
2. Consideration of the wider environmental context and interactions between socio-economic and political factors
3. Diseases or health conditions interact through reinforcing risk pathways.

Based on a review of the literature, Tsai^15^ proposed three models of disease interaction (the third syndemic criterion listed above):

1. Diseases or health conditions are mutually causal
2. Serially causal epidemics, where one health risk leads to another
3. Synergistically interacting epidemics, where the health burden is greater for each risk in the presence of the other^15^.

## Does a syndemic framework explain ethnic inequality and multimorbidity in psychosis?

Syndemic theory is evolving, and its application to psychosis requires careful adaptation. We now explore the evidence for each syndemic criterion in relation to ethnic inequality in psychosis and its comorbidity with other conditions to assess the extent to which this is an appropriate and helpful framework. For the purposes of this article, we will discuss the psychosis-diabetes comorbidity as an exemplar, especially given the mortality gap of up to 15 years observed in psychosis which is largely due to cardiovascular diseases such as diabetes^16^.

### Criterion 1: Two or more diseases or health conditions clustering within a particular population

Psychiatric conditions are diagnosed on a syndromal basis, namely by specific symptom patterns which lead to trajectories of progression and remission. There are biological correlates of psychosis diagnoses, although these are not invariably present. Psychoses encompass a diverse range of disorders with different levels of susceptibility influenced by both environmental and genetic factors. Disabling sub-threshold disorders, called psychotic experiences, for example, young people who are clinically at high risk for psychosis and present with attenuated psychotic symptoms are not necessarily associated with future conversion to psychotic disorders (only 25% over 2–3 years^17^). Thus, psychotic symptoms exist on a continuum and interact with non-psychiatric conditions in more complex ways than two discrete biomedically defined conditions.

People with psychotic disorders are more likely to experience multimorbidity than those without psychotic disorders^18,19^. Extant literature has shown that the prevalence of diabetes in psychosis exceeds that in the general population, with prevalence rates in psychosis ranging from 1.26 to 50% across studies (median 13%)^20^, whereas in the general population, the prevalence of diabetes for all age groups worldwide was estimated to be 2.8% in 2000^21^. Whilst the metabolic risks associated with the use of antipsychotic medication may explain this prevalence, meta-analytic findings report significantly impaired glucose tolerance and insulin resistance in patients with first-episode psychosis who are either antipsychotic-naïve or have minimal antipsychotic medication exposure^22^. The onset of psychotic experiences that predate the onset of physical health conditions have also been documented^23^. Clearly, research is urgently needed to understand the origins of the overlap between psychosis and diabetes as well as other physical illnesses to inform preventive practice and improve the life expectancy in this population.

### Specific populations or communities

Some migrants experience an increased risk of schizophrenia and other psychotic disorders compared with the majority population in given settings^24^. This risk extends to their descendants, with second-generation immigrants also at elevated risk for psychosis, although this varies by ethnicity^25^. Risk correlates with visible minority status, meaning that Black Caribbean and African migrants and their descendants in Europe and North America experience the greatest incidence^26^. Rates are also 2–4 times higher in people of Pakistani and Bangladeshi origin in the United Kingdom^27^ and Moroccans in the Netherlands^28^.

Furthermore, higher multimorbidity rates are observed in ethnic minority communities that experience psychosis. For instance, Black people with a psychotic disorder are at a higher risk of type 2 diabetes^29,30^. The origins of this multimorbidity are not clear and could cover a spectrum of factors. Examples include cultural, race and ethnicity-related risk factors such as interpersonal and societal racism and prejudice.

Some studies suggest that severe mental illness emerges with factors relevant to race and ethnicity-related health disparities to drive physical illnesses^16^. It is important to note that ethnicity also includes social and psychological influences, including identity, explanatory models and belief systems about health and illness, levels of social support and social assets, and health risk behaviours which may also vary across ethnic groups. These may influence help-seeking, selection of preferred care providers, early recognition and intervention and recovery. Variations in these processes may explain ethnic inequalities of experiences and outcomes. Essentially, the relationship between psychosis and physical health conditions such as diabetes is not linear but rather a result of the interaction between multiple risk factors and conditions, with multiplicative or additive effects on outcome^16^.

### Criterion 2: Consideration of the wider environmental context and interactions between socio-economic and political factors

Emerging evidence suggests the underlying pathophysiology of psychosis depends as much on macro-level factors as it does on one’s biology. Below we present the evidence for two macro-level factors which are likely to be most pertinent to the psychosis-diabetes comorbidity as an illustration.

### Discrimination

Discrimination as a mechanism involved in the pathway between minority status and psychosis vulnerability would also account for the increased risk observed across diverse minority groups^31,32^. Research has shown that major experiences of discrimination are associated with an increased risk of diabetes, independent of obesity or behavioural and psychosocial factors^33^. Whitaker and colleagues investigated whether self-reported experiences of discrimination are related to diabetes over a 10-year period in a population-based cohort including four racial/ethnic groups. They observed major experiences of discrimination were associated with a greater risk of incident type 2 diabetes. Interestingly, the association between major experiences of discrimination and diabetes was present regardless of whether the discrimination was attributed to race/ethnicity or to other causes. Therefore, a relationship between discrimination and comorbid diagnosis of psychosis and diabetes is highly plausible, given what is known about the underlying complex mechanisms that drive these conditions.

### Urbanicity

Studies have shown elevated rates of psychotic disorders associated with densely populated areas^34^, urban birth^35^ and current city living^36^. Indeed, the lifestyles stemming from these environments contribute to diabetes risk. Such areas may contain large amounts of fast-food facilities, poor access to quality foods, lack of health care resources, and promote social isolation. This context then encourages participation in adverse health behaviours such as unhealthy eating, physical inactivity and excess weight gain. A recent study reported a statistically significant association between severe mental illness and type 2 diabetes comorbidity and neighbourhood-level socio-economic disadvantage^37^.

### Criterion 3: Diseases or health conditions interact to result in adverse disease interaction which worsens health outcome

Burgeoning evidence over the past decade consistently demonstrates that the interaction between social, psychological, and biological risk factors can provoke psychotic symptoms with the exact combination of causes different for each person. Most studies conclude that the excess of poor health outcomes in marginalised communities is partially attributable to the cumulative effects of risk factors at a particular point in time but also over the life course.

Extant literature has shown a relationship between adverse childhood experiences (ACEs) and psychosis in adulthood. ACEs are a broad construct covering a range of experiences, including abuse, neglect, and exposure to domestic violence^34^. ACEs are robust predictors of psychosis^38^ and physical illnesses such as diabetes^39^, with differential distribution of adversities across race and ethnicity^40^. Adversity appears to operate alongside and possibly through biological pathways, especially in the stress response system^41^. Research shows exposure to ACEs increases the odds of medical morbidity by up to eight times among people with bipolar disorder^42^. Though to our knowledge, no study has investigated the link between (1) ACEs, diabetes and psychosis; (2) ACEs, diabetes, ethnicity and psychosis.

## Discussion

Syndemic theory is very much about specific populations in certain places of highly clustered disadvantage and life histories of multiple adversities colliding to create the conditions in which bio-psycho-social interactions lead to multiple co-occurring conditions. We propose the most important implications of syndemic research to psychosis, particularly the ethnic inequality and multimorbidity in psychosis, are: (1) to understand the complex aetiological interplay of multiple sources of adversity, and (2) to consider prevention and interventions that operate at multiple levels addressing multiple risk factors and their synergy. The syndemic framework fits the idea that there are multiple ecological and social drivers of psychosis and its comorbidity with other health conditions—an idea borne out by what is seen in clinical practice and the emerging literature. Prevention and care should reflect this.

### Challenges to applying syndemic theory to psychosis

It is important to note that psychosis is a set of symptoms and can be triggered by other conditions, such as drug use. In fact, symptoms and risk factors are themselves conditions (e.g., psychotic symptoms and poverty), and behaviours can also be considered conditions (e.g., smoking, drug and alcohol use). Therefore, conditions and behaviours are used interchangeably in the literature^15^.

Another challenge to applying syndemic theory to psychosis is that statistical methods to test synergistic and additive effects need large samples, but psychosis is rare^43^, and there are challenges to pool sufficient people with psychosis across datasets and places to test these theories.

### Summary of research to date

Against this background, we contend that ethnic inequalities and the multimorbidity present in psychosis can be further understood through a syndemic framework. Although this paper has used diabetes as an illustration, it is one of the many examples that could be taken into consideration. We suggest empirical studies to test the framework, which may explain health inequalities among specific populations over-represented in psychosis (minoritized communities) with historical and contemporary adversity as determinants of multimorbidity related to psychosis and consequent premature mortality.

### Future research directions

#### Specify contexts

Currently, studies tend to investigate macro- and micro-level factors independently, and to advance the field these need to be examined in combination. It is vital to document the context in which a proposed comorbidity or multimorbidity is observed. In syndemic models, this context is what drives the association between the conditions of interest. If an association between two health conditions occurs at the same prevalence across all populations, then it is not a syndemic but a common universal comorbidity. However, if the rates of occurrence vary by context, then there is something about the risk factors in that context which may constitute a syndemic. There needs to be a clear understanding of the social, cultural, political, economic, geographic and environmental aspects of that context.

#### Combine quantitative and qualitative methods

Currently, literature concerned with psychosis increasingly employs quantitative and statistical methods. Future studies should aim to include qualitative, mixed, or multi-method approaches to truly uncover the complex aetiologies of psychosis and its comorbidities. In particular, in-depth narratives can expose previously unknown variables and more complex relationships between contexts and individual characteristics.

#### Propose a mechanism for action for the syndemic interaction

A syndemic requires a proposed pathway from the social context to the interaction of two or more health conditions. Clearly articulating the hypothetical mechanism underlying the interaction allows for appropriate study design and analysis to test the pathway, which will consequentially permit the design of effective prevention and intervention syndemic care models. For example, inflammatory mechanisms can link the social environment, health behaviours, and psychological and biological processes.

## Conclusion

Existing evidence shows that there are social determinants (including structural factors) that, independently or in synergy, raise the risk of illnesses generating multiple illnesses. But these factors also operate to prevent recovery as well as generate and sustain harm in society, ultimately compounding and worsening inequality.

Recognition of psychosis as a candidate for a syndemic framework provides an avenue for novel public health and clinical research that is attentive to the multiple dynamics at play in health. A syndemic approach offers a bio-psycho-social framework that accounts for context-specific populations and suggests the need for new strategies to both improve public health and treat individual patients. These systems of interventions must show alignment across policy and practice with considerable tailoring to the places and contexts in which a syndemic might emerge.
